# Measuring Neural Arousal for Advertisements and Its Relationship With Advertising Success

**DOI:** 10.3389/fnins.2020.00736

**Published:** 2020-07-15

**Authors:** Esther Eijlers, Maarten A. S. Boksem, Ale Smidts

**Affiliations:** Department of Marketing Management, Rotterdam School of Management, Erasmus University Rotterdam, Rotterdam, Netherlands

**Keywords:** EEG, advertising, arousal, consumer neuroscience, neuromarketing, attitudes

## Abstract

Abundant research has established the important role of ad-evoked feelings on consumers’ reaction to advertising. However, measurement of feelings through explicit self-report is not without its limitations. The current study adds to previous work by showing a sophisticated way of first estimating how arousal is represented in the brain via an independent task (using EEG), and thereafter using this representation to measure arousal in response to advertisements. We then estimate the relationship between the identified process (arousal) and external measures of ad effectiveness (as measured by notability and attitude toward the ad). The results show that the neural measure of arousal is positively associated with notability of ads in the population at large, but may be negatively associated with attitude toward these ads. The implications for the application of EEG in ad testing and for understanding the relationship between arousal and effective advertising are discussed.

## Introduction

Advertising is an important means for companies to convey to consumers what they have to offer and thereby brings together supply and demand. The successfulness of advertising is an important factor in increasing the sales of products and services. Multiple marketing research techniques are currently applied to develop effective ads and to assess their effectiveness. These methods range from self-report measures and focus groups to the more innovative autonomic measures and brain imaging techniques in small so-called “neural focus groups” (e.g., Poels and Dewitte, 2006; Ariely and Berns, 2010). In the present study, we specifically investigate the impact of arousal in response to advertisements as measured with EEG and assess the relationship between this “neural arousal” measure and measures of advertising effectiveness in an independent larger sample of the population.

For companies, it is important to assess consumers’ responses to advertisements before spending substantial amounts of money on the broadcasting of an advertisement. These responses include consumers’ judgments or cognitions about advertisements, but, critically, also consumers’ first reactions and “feelings” in response to ads are deemed to be important (e.g., Burke and Edell, 1989). Abundant research has established that ad-evoked feelings are strong predictors of consumers’ response to advertising (Edell and Burke, 1987; Holbrook and Batra, 1987; Stayman and Aaker, 1988; Burke and Edell, 1989; Olney et al., 1991; Morris et al., 2002; Pham et al., 2013).

An important component of these ad-evoked feelings is arousal (e.g., Holbrook and Batra, 1987). Arousal is a fundamental aspect of emotion and is defined as the intensity or level of activation of one’s (emotional) response (Lang and Bradley, 2010). Over the years, multiple studies in advertising have shown a positive relationship between measures of arousal and advertising effectiveness such as attitude toward the ad (Holbrook and Batra, 1987; Olney et al., 1991), attitude toward the brand (Holbrook and Batra, 1987), purchase intention (Morris et al., 2002), and viewing time (Olney et al., 1991).

In all the above-mentioned studies, consumers’ feelings in response to advertisements have been measured using self-reports. Although certainly not without merit, such reports, particularly when they involve reporting on feelings or other internal states, do have their limitations. In addition to the fact that people are limited in their ability to reflect accurately on their internal mental processes, concerns with social desirability compound this problem (Nisbett and Wilson, 1977; Wiles and Cornwell, 1990). Moreover, the ability to report on mental states requires cognitive processing, which may interfere with or even change the originally evoked feelings (Poels and Dewitte, 2006). Although the use of non-verbal graphical scales such as the well-known Self-Assessment Manikin (SAM) may reduce the amount of cognitive processing required (e.g., Morris et al., 2002), it still requires introspection and reflection on mental processes which largely occur outside our awareness (Zajonc, 1980).

Implicit techniques that measure physiological consequences (of psychological antecedents) in response to advertisements, share the advantage that they are free of the cognitive biases described above. A relatively new development in marketing is the application of brain imaging to measure the direct implicit response of the brain toward products, brands and advertisements (Plassmann et al., 2015). Electroencephalography (EEG) is the most popular of these methods in neuromarketing practice, particularly in ad testing (Smidts et al., 2014), because of its relatively low costs and high temporal resolution compared to, for example, functional magnetic resonance imaging (fMRI) (Ariely and Berns, 2010).

EEG is a non-invasive method to record brain activity by means of measuring voltage changes at the scalp. Decades of research have shown that oscillations in the EEG signal in certain frequency ranges can be associated with specific psychological processes in the brain (e.g., Basar et al., 1999). One of these frequency ranges, or frequency bands, in the EEG signal is the alpha frequency band which is defined as oscillations between 7 and 12 Hz. Desynchronization of the alpha band or “alpha suppression” in response to opening the eyes has been observed since the first application of EEG by Berger (1929), see also Barry et al. (2007). EEG studies investigating arousal showed that the processing of emotional arousing stimuli is related to alpha suppression (e.g., Simons et al., 2003; DeCesarei and Codispoti, 2011, but see Aftanas et al., 2002; Uusberg et al., 2013).

The generally adopted procedure in experimental studies investigating emotional arousal presents participants with stimuli from the International Affective Picture System (IAPS, Lang et al., 2008) (e.g., Müller et al., 1999; Keil et al., 2001; Aftanas et al., 2002; DeCesarei and Codispoti, 2011; Uusberg et al., 2013). These standardized and validated photographs depict various scenes, objects and people, and have been shown to evoke emotional states varying along the two dimensions of valence and arousal. Viewing arousing stimuli enhances cortical excitation compared to viewing neutral or less arousing stimuli, and thereby reduces alpha activity (DeCesarei and Codispoti, 2011).

Whereas measuring this type of brain activity and inferring a specific psychological process from this measurement is simple and straightforward, it is also problematic because it involves reverse inference (Poldrack, 2006; Lee et al., 2017). The fact that inducing high arousal leads to observing alpha suppression (forward inference), does not warrant the reverse inference that observing alpha suppression in a particular instance must mean that arousal is high. This logic is only valid if arousal is the only process that would lead to alpha suppression, which is not the case since also attention, memory demands and general alertness suppress alpha oscillations (Klimesch, 2012). Furthermore, also other frequency bands have been related to arousal, for example increased gamma band activity (30–65 Hz) has been observed in response to emotional arousing pictures compared to neutral pictures (Müller et al., 1999; Keil et al., 2001). This so-called reverse inference problem thus undermines most of the techniques used in neuromarketing practice today.

In the current study, we therefore first applied a so-called “functional localizer task” to search for patterns of EEG oscillations that we could objectively and independently estimate via an independent task as being associated with arousal. We adopted the same procedure that is generally used in other studies investigating emotional arousal, hence we presented the participants with IAPS stimuli. The oscillatory EEG activity evoked by the arousal dimension of these pictures will be used as the measure of arousal.

In addition, although EEG can be fruitfully applied to measure the implicit arousal response to advertisements, it is crucial to show that the inferred psychological process is actually relevant for advertising. We therefore investigated whether arousal evoked in response to the ads, as measured neurally in a relatively small group of individuals, is associated with measures of advertising effectiveness in the population at large. Even though EEG is currently the main method applied in neuromarketing practice, evidence on the relationship between EEG oscillations and measures of advertising effectiveness is limited (Chang, 2017). In only a handful recent studies, EEG-based measures have been shown to be related to market-level success (Boksem and Smidts, 2014; Dmochowski et al., 2014; Venkatraman et al., 2015; Barnett and Cerf, 2017). While the aim of the current study is not predicting market success, it would add to this previous work by showing the specific role of arousal in response to advertisements, as inferred from an EEG measure estimated via an independent task in one and the same study, in relation to measures of advertising effectiveness in a separate, larger sample of the population.

Advertising effectiveness can be defined in multiple ways, since there are several different communication objectives for advertising. A first communication objective is increasing awareness in consumers, in order to be able to rely upon memory, either recall or recognition, before or at the moment of the purchase decision (Percy and Rossiter, 1992). Research on personal theories of practitioners in advertising revealed that professionals in the field see capturing consumers’ attention as their main goal in order to break through “the clutter” of other advertisements and daily life (Kover, 1995; Nyilasy et al., 2013). A second objective of advertising is generating a favorable attitude toward the ad because of its influence on brand attitude (MacKenzie et al., 1986; Brown and Stayman, 1992) and subsequent behavior (Ajzen and Fishbein, 1977). In the present study, we obtained ratings from a large sample of the population on notability and attitude toward print advertisements as measures of advertising effectiveness covering these two communication objectives (awareness and attitude toward the ad).

In sum, in the present study we first applied a localizer task in order to functionally localize arousal-related patterns of oscillatory EEG activation. After having estimated how arousal is represented in the brain, we measured arousal evoked by print advertisements, and related those neural measures of arousal to measures of ad effectiveness in the population, consisting of self-reported ratings of notability and attitude toward the ad.

## Materials and Methods

### Participants

The sample for the EEG study consisted of thirty-one students (16 female, age range = 19–27 years, *M* = 22.3, SD = 2.5) recruited from the university population. They all had normal or corrected-to-normal vision and had no history of neurological illness. In return for their participation they received 30 Euros.

The population sample consisted of an external consumer panel of 1,260 participants of the same nationality as that of the EEG participants (650 female, age range = 18–88 years, *M* = 54.1, SD = 12.4). These participants were randomly drawn from a consumer panel that is representative of the Dutch adult population (18 years and older), by a certified market research firm. Their educational background covered the whole range from low (23%), to intermediate (35%), to high (42%).

### Tasks

#### EEG Functional Localizer Task

In this task, we showed our 31 participants 100 standardized and validated photographs from the International Affective Picture System (IAPS, Lang et al., 2008) while we recorded their EEG, in order to functionally localize arousal-related patterns of activity. We selected photographs at the low and high ends of the arousal dimension, and at the negative and positive ends of the valence dimension in order to keep a balanced design and to control for valence of the stimuli. This results in four picture categories, each containing 25 photos: positive valence/low arousal, negative valence/low arousal, positive valence/high arousal, and negative valence/high arousal. Averaging the mean normative ratings on arousal and valence of all our selected pictures results in the following mean ratings per picture category (on a scale from one to nine): positive valence = 7.32/low arousal = 3.56, negative valence = 3.56/low arousal = 3.61, positive valence = 7.12/high arousal = 6.52, negative valence = 2.73/high arousal = 6.75 (see Supplementary Appendix 1). Stimuli were presented for 3,000 ms, with an interstimulus interval of 1,500 ms. We instructed participants to empathize with the situation depicted and try to imagine being in that situation.

#### Print Advertisements

The stimuli in this task consisted of 150 real print advertisements from the United States from the Starch database (GfK MRI United States) and Ads of the World, in order to guarantee that they were new to our European participants and thus to control for familiarity of the ads. The ads pertained to five product categories: cars, gadgets, food, beauty, and fashion. Only women viewed the beauty and fashion advertisements, and only men the cars and gadgets advertisements. Both women and men viewed the food advertisements. During the EEG study, the advertisements were presented for 5,500 ms with an interstimulus interval of 1,500 ms. Brain activity was recorded while participants viewed the advertisements.

Participants from the population sample rated a random subset of 10 ads on two measures of ad effectiveness as part of an online survey: *Notability of the ad*, and *Attitude toward the ad* (nine items averaged), in order to capture effectiveness of the advertisement in the population at large (for more details on the items, see Supplementary Appendix 2). We created one measure of *Attitude toward the ad* out of the nine items, because the items mutually correlated significantly at the level of *p* < 0.01 (ranging from *r* = 0.59 to *r* = 0.93), Cronbach’s α was .96, and a principal component analysis (PCA) returned only one component explaining 84% of the variance. All items were Z-transformed at the advertisement level. The descriptives of the raw scores at the advertisement level indicated that the 150 ads differed substantially both in Notability and Attitude (see Table 1).

**TABLE 1:** Descriptives of raw scores on notability and attitude toward the ad at the advertisement level (5-point scale).

	*M*	SD	Min	Max
Notability	3.51	0.32	2.77	4.11
Attitude	2.90	0.27	2.22	3.52

Since the population sample is more diverse in age and level of education than the EEG sample, we checked whether a subsample of the population sample with similar characteristics as the EEG sample (relatively young and highly educated) differed in their ratings of the print advertisements from the overall sample. Ratings appeared to be robust to differences in age and education (see Supplementary Appendix 3).

### EEG

#### Recording

The EEG data was acquired using the BioSemi Active Two system with 64 active Ag-AgCl electrodes. Additional flat type electrodes were placed on the right and left mastoid, and in the eye region in order to record eye movements or electro oculograms (EOGs). Electrodes were placed below and above the left eye in line with the pupil to record vertical EOGs, and at the outer canthi of both eyes to record horizontal EOGs. The EEG and EOG signals were sampled at a rate of 512 Hz, and digitally low-pass filtered with a 128 Hz cut-off (3 dB).

#### Preprocessing

All preprocessing was done in Brain Vision Analyzer software (BVA; Brain Products). The data was first down-sampled to 256 Hz, then re-referenced to the averaged mastoids, and filtered with a low cutoff filter of 1 Hz and a notch filter of 50 Hz with a slope of 48 dB/octave. We split the continuous data into 100 segments (one for each IAPS picture) for the functional localizer task, and 90 segments (one for each ad) for the print ad task, with segments lasting from stimulus onset until 2.5 s after stimulus onset. Then, we applied Gratton and Coles ocular correction to correct for eye movements as implemented in BVA, and a standard artifact detection and rejection procedure in which segments were rejected that contained jumps larger than 30 μV/ms, amplitude differences exceeding 150 μV/200 ms, and amplitude differences below 0.5 μV/100 ms. Note that only the channels that contained artifacts were deleted within the given segment, and not the entire segment. For all segments, we decomposed the signal into components of frequencies ranging from 1 to 128 Hz using a Fast Fourier Transform (FFT) (BVA, using a 10% hamming window). The resulting spectral data was exported to Matlab (Mathworks).

#### Data Reduction

We then estimated how (at which frequencies in the signal) and where (at which electrodes on the 64-channel cap) the difference between low and high arousal is most pronounced across participants. To achieve this, we tested on the first (participant) level, whether the affective category that the presented picture belonged to could be used to predict the EEG data. To this end, we first log transformed the EEG data for normalization purposes. We then regressed the EEG data onto three independent variables (IV’s): arousal, valence, and the interaction of valence and arousal, respectively. Even though our main interest is arousal, for completeness, we also checked the differentiation in brain activity for valence, and for the interaction between valence and arousal. Per participant, the regressions were performed at each electrode and each frequency.

**FIGURE 1:**
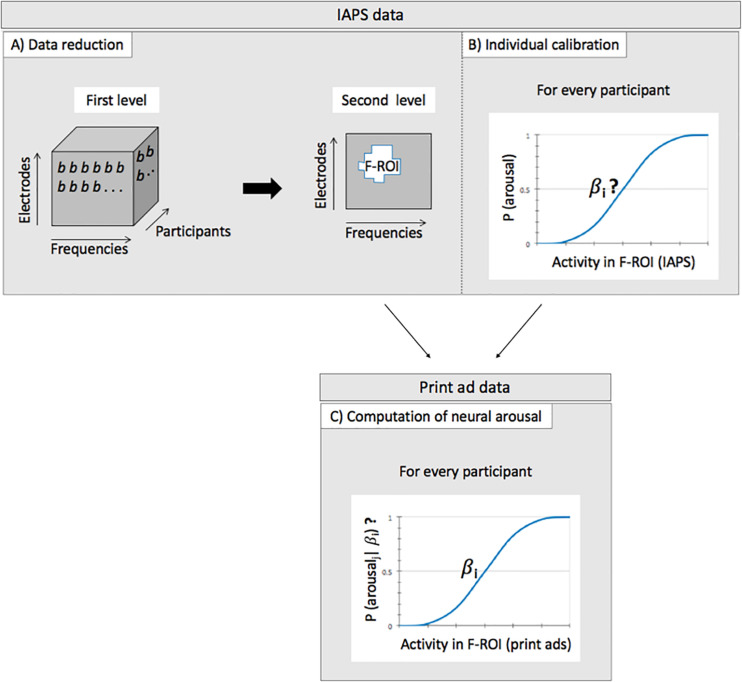
Schematic representation of the different steps of the analysis of assessing neural arousal per ad. Step **(A)** and step **(B)** are performed on the IAPS data, step **(C)** on the print ad data. **(A)** Data reduction by searching for how and where in the brain, the difference between low and high arousal is consistently represented in the EEG data across participants. This step results in an arousal Frequency by Region of Interest (F-ROI). **(B)** Individual calibration of the data as defined in panel **(A)**, by searching for each participant for the optimal relation between the participants’ specific EEG response on the one hand (in the F-ROI) and the IAPS pictures on the other hand (DV = low vs high arousal; logistic regression). This step results in participant specific coefficients (indicated by β*_*i*_*) that link EEG activity and arousal level. **(C)** Computing for each participant *i* and print ad *j*, the probability that a print advertisement evokes neural arousal by selecting activity from the arousal F-ROI that is obtained while viewing the print ads (and given β*_*i*_*).

Then, at the second level (i.e., the group level), the resulting regression coefficients (i.e., one for each electrode-frequency combination) were used to test the overall arousal effect across participants. Here, we searched for a cluster of frequencies and electrodes where the regression coefficients consistently deviated from zero across participants using (one-sample) *t*-tests. That is, we searched for activity that represents a difference between low and high arousal states in a similar fashion across participants (Step A in Figure 1). More specifically, we use a cluster-based permutation test [from the Mass Univariate ERP Toolbox; (Groppe et al., 2011)] to search for how and where on the scalp, the difference between low and high arousal is consistently represented in the EEG data across participants. The reported *p*-values are corrected for multiple comparisons via this permutation test. The test is based on a “cluster mass” statistic, and the output consists of a cluster of data points (frequencies, electrodes) where an effect is present across participants, with a family-wise-error corrected *p*-value for that cluster, thresholded at *p* < 0.05. The result of this analysis is a cluster of frequencies and electrodes where the activity represents a difference between low and high arousal states in a similar fashion across participants. We will therefore refer to this this cluster as the arousal Frequency by Region of Interest (F-ROI).

Using this method, we obtained a cluster of frequency-electrode combinations at which the EEG activity evoked by high and low arousal images differed significantly, in the alpha frequency band (7–12 Hz) on central electrode locations (see section “Results”). No such robust effects of valence (main effect and interaction with arousal) were observed so the valence dimension will not be discussed further. Since the literature on valence often reports asymmetry-effects (e.g., Davidson, 2004), we additionally transformed the data to specifically search for asymmetrical effects where we subtracted for each electrode, data at the electrode at the exact opposite side of the other hemisphere (except for the midline electrodes). We did not find additional significant clusters based on this transformed data.

#### Calibration

After having defined how arousal is represented in the brain across participants, we conducted individual calibration on this data to take heterogeneity of the brain response into account (Step B in Figure 1). Although we found a similar *pattern* of brain activity in response to the low versus high arousal pictures across participants in step A, this does not necessarily mean that the specific/exact EEG response to the low and high arousal pictures is the same across participants. Differences in properties of the skull, in the orientation of the neuronal sources and coherences between the sources, in addition to differences between responses at the neuronal source, result in heterogeneity of EEG responses across participants (Teplan, 2002). We conducted the individual calibration by performing a logistic regression for every participant. We took the (Z-transformed) EEG data from the arousal F-ROI, averaged across the selected frequencies and electrodes, to serve as independent variable, and the IAPS picture category as the dependent variable (DV: low versus high arousal). The participant specific coefficients that resulted from the logistic regressions can be used in the next step of the analysis on the EEG data obtained while viewing the print ads, in order to scale the EEG responses to the ads, to the standardized and validated levels of arousal of the IAPS pictures.

#### Estimating Arousal in Print Ads

In the next step of the analysis pertaining to the print advertisements, the EEG data that was recorded while viewing the print ads was also first transformed to the frequency domain. We then selected EEG data from the arousal F-ROI as determined in the first step of the analysis, but measured while viewing the print ads, for every participant. In addition, we scaled this activity in response to the ads for every participant, to the arousal level of the standardized and validated set of IAPS pictures. We did this by using a logistic function. For every participant, the selected (Z-transformed) activity from the arousal F-ROI was averaged across the electrodes and frequencies to serve as a predictor. We then used the participant specific coefficient that was computed in the second step of the analysis and a logistic function, to estimate the probabilities that the advertisements evoked arousal for that participant, based on the EEG activity in the F-ROI (Step C in Figure 1).

Finally, per ad, the estimated arousal probabilities were averaged across participants to arrive at a neural arousal score for each print ad, thus making the advertisement our focal unit of analysis.

Importantly, it should be noted that the individual calibration was performed to take heterogeneity in the EEG response to a specific print ad across multiple participants into account. That is, the same EEG response to a specific print ad, will indicate more arousal for this ad if it originates from someone who reacts *less* to the high arousing IAPS pictures, than when it originates from someone who reacts *more* to these same high arousing IAPS pictures. When someone is less easily aroused in general, and thus responds “with less arousal” to high arousing pictures, without individual calibration, this would lead to rather flat arousal responses in reaction to all print ads. Since our focal unit of analysis is the advertisement, we believe it therefore makes sense to scale the individual responses to these ads, to the validated set of IAPS pictures. Not conducting this individual calibration would result in less clean (more crude) relationships between the EEG activity and the arousal process that it should represent across individuals.

### Statistical Analysis

To test whether the neural arousal scores of the print advertisements are related to measures of ad effectiveness in the population at large, we computed the correlation between the neural arousal score of the ads, and averaged ratings of the ads by the population sample on Notability and Attitude.

To control for other features of the ads, we performed hierarchical regressions. We controlled for low-level visual features [i.e., luminance (i.e., the average pixel intensity) and contrast (i.e., the standard deviation of pixel intensity), using the “imread” function from the Image Processing Toolbox in Matlab] of the ad, brand familiarity, and dummies for product category in the first block, and tested the significance of the neural arousal scores in the second block. If one of the ad features other than the neural arousal scores appeared to be significant as a predictor, we also tested the interaction term consisting of that ad feature and the neural arousal scores in the third block.

Although we selected United States advertisements because they were new to our participants, several brands in the advertisements may have been familiar to the participants to varying extents. In order to control for the effect of brand familiarity, we collected data on “familiarity with the brand” with the brand logo presented and scale ranging from (1) *Very unfamiliar*, to (7) *Very familiar*, from a separate sample of 46 students, but with highly similar characteristics to the students in our EEG study (23 female, age range = 18–30 years, *M* = 21.0, SD = 2.6). Ratings were averaged across participants in order to arrive at one score per ad on brand familiarity (*M* = 4.6, SD = 2.0, range = 1.2–6.9).

## Results

### Functional Localizer

**FIGURE 2:**
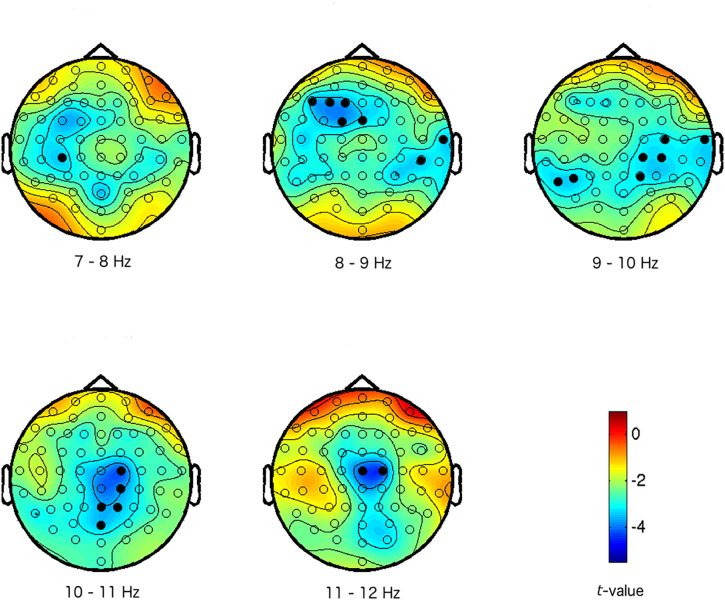
Maps of the difference between high and low arousal IAPS pictures in activity in the alpha frequency band (7–12 Hz). The colors represent *t*-values. The different scalp maps show the contrast (expressed in *t*-values) between high and low arousal for the specific frequencies that are mentioned below the maps, and across the head for the 64 electrodes. Electrodes with activations above the threshold are marked in black (i.e., electrodes that are part of the arousal effect/cluster/F-ROI: preset cluster inclusion for data points *p* < 0.005, cluster significance *p* < 0.01 FWE).

In the data reduction step of the functional localizer task, the analysis (Step A in Figure 1) reveals a significant difference between low and high arousal IAPS pictures that was consistently represented in the EEG data across participants (*p* < 0.01, FWE corrected). The effect was widespread but mainly present at central sites of the head, and in the alpha frequency range (7–12 Hz, on electrodes F1, F3, F5, FCz, FC1, Cz, C2, C4, T8, CP2, CP3, CP4, CP6, Pz, P2, P5, P7, and POz; see Figure 2). Activity in the alpha frequency range was lower in the high arousal condition than in the low arousal condition. This cluster of activity will constitute the arousal F-ROI.

### Relationship Between Neural Arousal and Population Sample Evaluation

**FIGURE 3:**
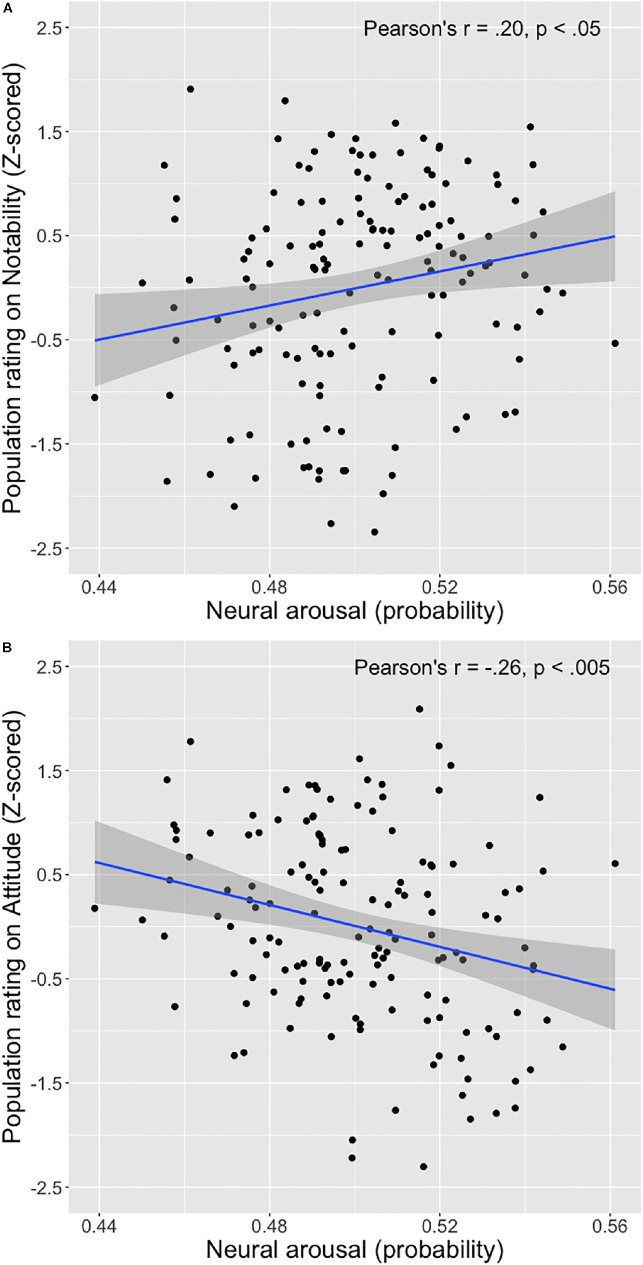
Scatterplots of the relationship between neural arousal score of the 150 print advertisements and average population sample ratings on notability of the ad **(A)** and attitude toward the ad **(B)**.

EEG activity selected from the arousal F-ROI that is described above, but obtained during viewing of the print ads was then used, together with the participant specific coefficient obtained in step B, to predict the probability that each specific ad evoked arousal (Step C in Figure 1). The resulting neural arousal scores of the 150 print advertisements were then correlated with the measures of ad effectiveness from the population sample. We observed a significant correlation with these measures of ad effectiveness: The higher the advertisements scored on neural arousal, the higher they were rated by the population sample on Notability (*r* = 0.20, *p* < 0.05), but the lower they scored on Attitude toward the ad (*r* = −0.26, *p* < 0.005, both *p*-values corrected for multiple comparisons) (see Figure 3).

When controlling for other ad features, these direct relationships between neural arousal and its respective ad effectiveness measures remain. Table 2 shows that neural arousal is positively associated with notability of the ad (standardized regression coefficient β = 0.19, *t* = 2.31, *p* < 0.05) when controlling for product category, brand familiarity and luminance and contrast. Adding neural arousal to the model in step 2 significantly increased the fit of the model (*R*^2^ change = 0.03, *F*-change = 5.33, *p* < 0.05). The interaction term between the neural arousal score and contrast was not significant (*p* > 0.10) and therefore did not enter the model. Similarly, Table 2 shows that adding neural arousal to the model predicting attitude toward the ad, significantly increased the fit of the model (*R*^2^ change = 0.06, *F*-change = 11.27, *p* < 0.005) with neural arousal being negatively associated with attitude toward the ad (β = −0.25, *t* = −3.36, *p* < 0.005). The interaction terms between the neural arousal score, dummy product category 1, dummy product category 2, and brand familiarity were all non-significant (*p* > 0.10) and did not enter the model.

**TABLE 2:** Relationships between population sample ratings on notability of the ad, attitude toward the ad, and features of the ad.

	Notability		Attitude	
	Beta	*t*	Beta	*t*
Product category cars vs food	–0.16	–1.51	−0.30***	–2.97
Product category gadgets vs food	–0.17	–1.58	−0.23*	–2.32
Product category beauty vs food	–0.17	–1.66	–0.08	–0.85
Product category fashion vs food	–0.11	–1.03	–0.12	–1.29
Brand familiarity	0.12	1.30	0.42***	5.10
Luminance	–0.09	–1.06	–0.03	–0.45
Contrast	−0.24*	–2.93	–0.04	–0.51
Neural arousal	0.19*	2.31	−0.25***	–3.36
*R*^2^ full model	0.13		0.23	
*R*^2^ change due to neural arousal	0.03		0.06	
*F* change due to neural arousal	5.33*		11.27***	

*p < 0.05, **p < 0.01, ***p < 0.005. Beta is the standardized regression coefficient.

Cross-validation analyses confirmed that neural arousal also contributed significantly to the models in predicting ratings of a holdout sample, supporting the results that neural arousal positively predicts notability of the ad, but negatively predicts attitude toward the ad, controlling for product category, brand familiarity and luminance and contrast (see Supplementary Appendix 4).

Because of the null result for valence related activity (and the negative relationship between neural arousal and attitude), we wanted to rule out the possibility that our arousal measure is biased toward negative valence. Conducting an independent samples t-test on arousal activity in response to the IAPS pictures (i.e., activity that is averaged across the arousal F-ROI), for positively versus negatively valenced IAPS pictures, indicates that these pictures on average do not differ in arousal for all participants (no significant differences for any of the participants; range of *t*-values = −1.88 to 1.92, one sample *t*-test on *t*-values is n.s. *M* = 0.06, *t* = 0.35). This shows that there is no (negative) valence bias in our arousal measure.

### Follow-Up Exploratory Analysis on Negative Relationship Between Arousal and Attitude

In order to shed light on why we (unexpectedly) found a negative relationship between neural arousal evoked by the print ads and ratings on attitude by the population at large, we followed up on the results with an additional exploratory analysis. Inspecting the scatterplot illustrating this relationship (Figure 3B) suggests that the ads in the bottom right corner contribute heavily to the negative relationship between our measure of arousal and ratings on attitude: More ads are present in this corner compared to in the other corners, and they extend more to the limits of the two variables. Examining these ads for a common denominator reveals that many of those ads deal with the human body in a creative, uncommon manner or contain unusual content of a physical nature, which may not be appreciated by some of our participants and may therefore not be perceived as positive. An example of such an ad depicts one arm with hand, with a second hand pulling of the skin of the first hand, as if an invisible glove is put on, advertising for a hand sanitizer. In another such ad, the face of a person is presented with a fist appearing from the neck, punching the person in the face, referring to the (wasabi) taste of the potato chips. We hypothesize that the negative relationship between arousal and attitude in our study may exist because of these creative ads that may be perceived negatively. Searching the whole stimulus set for print ads that tick this box (*k* = 22) and excluding these from the analysis, results in a non-significant correlation between arousal and attitude of *r* = −0.13 (*p* = 0.15) for the remaining *k* = 128 print ads. Thus, these (mostly negatively evaluated) creative ads indeed contributed to the negative relationship between arousal and attitude, which is perhaps due to the nature of the creativity used here (with the use of the human body in an uncommon fashion).

## Discussion

### Main Findings and Contributions

We presented a study in which we showed how arousal, as measured using EEG, is associated with measures of advertising effectiveness. The aim of this work was twofold. The first aim was to measure arousal evoked by advertisements using EEG, by first estimating how arousal is represented in the brain via an independent task. Our second aim was to show that this neural measure of arousal was associated with actual measures of marketing effectiveness. We specifically investigated whether the evoked arousal in a relatively small group of individuals was predictive of both the notability of the advertisements, as well as consumers’ attitudes to the ads in the population at large, which are considered the primary communication objectives for advertising.

We first administered a localizer task to functionally localize arousal-related patterns of EEG activity in order to diminish the problem of reverse inference. We found that arousal-evoking IAPS pictures prompted a change in power in the alpha band in our participants, allowing us to define an EEG map of arousal. The observed representation of arousal is in agreement with previous literature on the neural correlates of arousal, showing a decrease in alpha activity when comparing high to low arousing stimuli. For example, using similar stimuli from the IAPS as we used in the current study, DeCesarei and Codispoti (2011) identified an effect of arousal that overlaps significantly with the results presented here: a decrease in alpha power (9–12 Hz), primarily over parietal electrodes. Similarly, Simons et al. (2003) found that arousing pictures, but also emotionally arousing movie clips, elicited decreased alpha power (8–13 Hz) over central and parietal electrode sites.

It should be noted, however, that such effects are not always observed. For example, Aftanas et al. (2002) presented participants with IAPS pictures inducing high, moderate and low arousal, and found that it was the *high* arousal pictures that elicited the strongest alpha increase over parietal electrodes. While participants in our study, as well as in the DeCesarei and Codispoti (2011) experiment, passively viewed pictures, they were required to rate the pictures in the study by Aftanas et al. (2002). Future research could reveal whether this difference between active and passive viewing may explain differences in associations between arousal and alpha power. Similarly, although some studies also report EEG effects of valence (e.g., Uusberg et al., 2013), these effects are also not always observed (Aftanas et al., 2002; Simons et al., 2003; DeCesarei and Codispoti, 2011), as was the case in the current study. In summary, we were able to extract a neural representation of arousal, reflected by changes in the EEG alpha band, that aligns well with previous findings.

After defining EEG activity that is representative of arousal using the localizer task, we sampled this EEG response obtained during viewing of advertisements (i.e., from the arousal F-ROI), which allowed us to estimate the level of arousal experienced for each of the print advertisements (calibrated to the participants’ EEG response to the standardized and validated levels of arousal of the IAPS pictures).

The results showed that the neural measure of evoked arousal in response to the ads was positively associated with notability of the print ads, but that attitude toward the ads was negatively associated with neural measures of evoked arousal in response to the ads. The negative relationships between neural arousal and attitude may be explained by creative executions of the ads that seem not to be perceived positively. Our exploratory analysis suggested that the use of the human body in an unusual fashion may have contributed to the negative relationship between neural arousal and attitude.

### Theoretical and Managerial Implications

The results of the present study showed that the neural measure of arousal evoked in response to the ads as measured using EEG was associated with two communication objectives of advertising, even after controlling for other ad features such as contrast, luminance, product category, and brand familiarity. Using this measure of arousal in response to advertisements may provide advertisers with more concrete and actionable insights on the mental state of the consumer. Equally as important, these results imply that the EEG measure reflecting arousal could provide advertisers with more guidance as to whether the ad design meets a particular communication objective, which may serve as input into decisions to optimize the ad. That is, advertisers need to consider the main objective of their campaign when designing ads since different measures of ad effectiveness may not align and may call for different ad designs. Ads that are neurally arousing may be effective in the sense that they are highly notable and thus enhance awareness, but may not necessarily be effective in terms of being positively evaluated.

The first communication objective of raising awareness was reflected by ratings on notability. As expected, the more arousing ads were found to be more notable as rated by the population sample. We conjecture that the downstream consequences of our “notability measure” are comparable to the ones of “Starch *noted* scores” as seen in older research (e.g., Lucas, 1960; Spotts et al., 1997). The Starch score indicates the extent to which an advertisement has been “noted” by viewers, so both this measure and our measure assess at least initial attention and give an indication of the extent to which advertisements can break through “the clutter” of other presented material. Whether such scores, however, actually measure recognition and thereby ad memory is debatable (Lucas, 1960; Spotts et al., 1997).

For the second communication objective however, we found somewhat surprising results: more arousing ads were evaluated less positively. In general, a clear association between arousal and attitude should not necessarily be expected, since arousal and valence are orthogonal in the circumplex model of affect. That is, both positively and negatively valanced stimuli could be associated with high arousal. However, advertisements are typically positive in valence, which is probably why previous work in advertising has associated high arousal with more positive attitudes toward ads or brands (Holbrook and Batra, 1987; Olney et al., 1991). The exploratory analysis suggested that the (mostly negatively evaluated) creative ads contributed to the negative relationship between arousal and attitude, which is perhaps due to the nature of the creativity used (with the presentation of the human body in an uncommon fashion). Thus, an ineffective (unusual, confusing, non-fluent) execution of creativity may result in affective responses that are negative in valence, which in turn contribute to a negative relationship between neural arousal and attitude toward the ad. Future studies are necessary in order to further investigate the specific process underlying the negative relationship between arousal as measured with EEG and ad effectiveness found in the current study, before this relationship can be generalized.

Yet, research on personal theories of practitioners in advertising revealed that professionals in the field see attracting attention as the most important aspect of advertising (Kover, 1995; Nyilasy et al., 2013), and thereby assume that attention grabbing ads will be evaluated positively. Advertising agencies nevertheless realize that crafting a creative ad that cuts through the clutter involves taking a risk. In addition, they feel that their clients fear creative advertising and often resist creative advertising solutions (Nyilasy et al., 2013). The results of our study suggests that the fear of clients may perhaps be justified and thus that advertisers need to have specific communication objectives from their clients. It is important to determine in advance how crucial it is, e.g., given the current status of the brand, to stand out and create brand awareness with the advertisement while taking the risk of not getting it attitudinally right.

### Limitations and Future Research Directions

We acknowledge several limitations that should be considered in the interpretation and generalization of our findings. First, while our approach of first estimating the neural substrate of arousal in an independent task mitigates the problem of reverse inference, it does not completely abolish it. Although in our opinion not very likely, it is still possible that a process other than arousal would have the exact same neural substrate as reflected in EEG recordings. Indeed, also attention, memory demands and general alertness have been found to suppress alpha oscillations (Klimesch, 2012), albeit with slightly different distributions across the scalp. Future research could try to separate these processes by using localizers not just for arousal, but also for attention and memory demands, for example.

As mentioned above, the negative relationships between arousal and attitude may be specific to the stimulus sets that were used here. It would therefore be valuable to study creativity in more depth, in order to search for patterns in executions of creativity that are perceived as positive versus negative. This knowledge will aid in the prediction of the actual tone of interest toward the ad (i.e., pleasurable or unpleasurable), in addition to the mere intensity of the interest response in the form of arousal.

While our measures of advertising effectiveness are important ones, more research is necessary to understand the specific downstream consequences of these measures. For example, even if more notable ads cause better memory of the ad, the memory for the brand or product will depend on many factors such as focus of the ad (on product or brand versus not), consistency between existing knowledge of the brand and the execution of the ad, familiarity of the brand, and possibly an interaction between these factors. In addition, the question remains how notability and attitude are weighted in determining the ultimate decision to purchase the product or not.

Finally, although we showed that our neural measure was associated with measures of advertising effectiveness at the population level, we did not examine if its contribution is above and beyond that of self-reports. This was not the aim of the current study, but it is important to emphasize because the question remains whether applying neuroimaging is more beneficial in predicting commercial success than other measurements such as questionnaires and to what extent this may be the case. Relatedly, it could be that neuroimaging methods other than EEG (such as fMRI) may be more strongly associated with effectiveness of advertisements or underlying psychological processes such as arousal (Chan et al., 2019, 2020); Venkatraman et al., 2015). Nevertheless, as we have shown here, neural measures may reveal important insight into the underlying processes (such as arousal) that may be associated with responses toward advertising messages.

## Final Conclusion

In summary, the current study demonstrates how to accurately measure arousal evoked in response to advertisements using EEG. Additionally, we show that this neural measure of evoked arousal as obtained in a relatively small group of individuals and estimated via an independent task in one and the same study is useful for marketing purposes. While controlling for other ad features, it contributes uniquely to explaining different measures of advertising effectiveness in the population at large that cover the main communication objectives of advertising. This evidence is important because of the increasing implementation of small so called “neural focus groups” in marketing practice, aimed at providing indices of advertising effectiveness at the population level. The findings suggest that different levels of alpha activity evoked in response to ads cannot automatically assumed to be associated with ad effectiveness in general. Although advertisements that evoke more arousal, or less alpha activity, will likely be more noted, they are not necessarily perceived positively. Nevertheless, using this EEG measure and taking the discussion points into consideration allows advertisers to improve on assessing the effectiveness of an ad design at an early stage, and even apply this measure on a moment-to-moment basis (Eijlers et al., 2019). Because of the relative inexpensiveness of EEG, the benefits of more and concrete improvements on ad designs, and the unique insights into the underlying psychological processes may indeed outweigh the costs.

## Data Availability Statement

The datasets generated for this study are available on request to the corresponding author.

## Ethics Statement

The study involving human participants was reviewed and approved by ERIM Internal Review Board (ERIM IRB), Rotterdam School of Management, Erasmus University. The participants provided their written informed consent to participate in this study.

## Author Contributions

EE, MB, and AS formulated the research question, designed the studies, and wrote the manuscript. EE collected and analyzed the data. All authors contributed to the article and approved the submitted version.

## Conflict of Interest

The authors declare that the research was conducted in the absence of any commercial or financial relationships that could be construed as a potential conflict of interest.
