# Supplementary material for: Measuring Neural Arousal for Advertisements and Its Relationship With Advertising Success
Source: Front Neurosci. 2020 Jul 15;14:736. doi: 10.3389/fnins.2020.00736 (PMC7378323; doi:10.3389/fnins.2020.00736)
Supplement: Supplementary file 1 [file Data_Sheet_1.docx]

**Supplementary Materials**

**Appendix 1**

Presented IAPS pictures:

positive valence/ high arousal 1650, 5621, 5626, 5629, 8030, 8034, 8080, 8161, 8170, 8178, 8179, 8180, 8185, 8186, 8190, 8191, 8200, 8251, 8300, 8341, 8370, 8400, 8470, 8490, 8501; positive valence/ low arousal 1604, 1610, 1620, 2299, 2304, 2360, 2370, 2387, 2388, 2530, 2540, 5000, 5001, 5010, 5200, 5201, 5220, 5551, 5611, 5760, 5779, 5780, 5811, 5891, 7325; negative valence/ high arousal 1050, 1052, 1120, 1300, 1525, 1931, 1932, 2730, 3500, 3530, 5971, 6230, 6250, 6260, 6300, 6313, 6350, 6510, 6540, 6550, 6560, 8485, 9600, 9800, 9810; negative valence/ low arousal 2221, 2399, 2440, 2490, 2491, 2590, 2722, 2750, 2753, 5120, 5130, 7060, 7224, 7234, 7700, 9000, 9001, 9046, 9090, 9220, 9280, 9290, 9330, 9331, 9360

**Appendix 2**

Each of the 1260 participants from the external consumer panel (650 females) rated 10 print ads, which results in 12600 ratings in total.

Items panel data (translated into English):

S01 Is notable

S02 Arouses my interest in the brand or product

S03 Stimulates me to respond or go to a store

***S04*** Is confusing and hard to understand (R)

S05 Makes me feel good

S06 Moves me

S07 Is joyful to watch

***S08*** Irritates me (R)

S09 Is believable

Response scale: 1 = Strongly disagree to 5 = Strongly agree

R = reverse coded

Overall rating

If you would have to rate the overall impression of the advertisement on a scale from 1 to 10, which score would you give? Response scale: (1) Very bad to (10) Excellent.

Performing a PCA on the ten items resulted in two extracted components with eigenvalues over Kaiser’s criterion of 1, with only Notability loading on the second component. Items S02, S03, S04, S05, S06, S07, S08, S09, and the overall score were taken together to form the effectiveness measure of Attitude toward the ad. S01 is the measure of Notability of the ad. The final measures Notability and Attitude do not correlate (*r* = -.11, *p-value* = .19).

**Appendix 3**

Our population sample consists of a random sample from the general population, which can be regarded as a strength of the study. However, the EEG data that is measured in response to the print ads is measured in a more homogeneous sample of university students. In order to establish that our results are not an artefact of the differences between the EEG sample and population sample we checked whether different subgroups in the population sample rated the ads in a similar fashion. Specifically, we inspected the ratings from a subset of the population sample with similar characteristics as the EEG sample with respect to age and education (n=62 out of the 1260, education: at least high school or higher and age: 18-35 years). We label this subgroup of the population the 'EEG-like sample'. Supplementary Figure 3.1 shows the average ratings from the total population sample and from the EEG-like sample on Notability and on Attitude.

*Supplementary Figure 3.1. Averaged ratings of the ads on Notability and Attitude by a subsample of the population with the same characteristics as the EEG sample, and by the total population sample. The central mark represents the median, with the edges of the box representing the 25th and 75th percentiles. The points outside the 25th and 75th percentiles are indicated as 'outliers'.*

The distributions of ratings on Notability and Attitude appear to be very similar for the two samples. Independent samples t-tests revealed no differences between ratings from the two different samples on Notability or Attitude (p = 1.0 for both measures).

In addition, we computed the correlations between the ratings on notability and attitude from the EEG-like sample and the total sample in order to check whether the same ads are rated as low and high on these measures. The correlations were highly significant (p < .001) with Pearson´s r = .55 for Notability and r =.49 for Attitude. Visual inspection of ratings per age group and per education type revealed similar patterns for all different subgroups. We therefore conclude that the findings in our study are not artefacts and not caused by differences between the two samples.

**Appendix 4**

In order to validate the use of the participant specific coefficients in the individual scaling of the EEG activity in response to the print ads, we checked whether the classification accuracies of the logistic regressions on the IAPS EEG data deviated from chance level across participants. This was indeed the case (*M* = 55.10%, *t* = 5.05, *p* < .00005). In addition to comparing the classification accuracy of the IAPS pictures to chance level, we applied permutations that confirmed that the class labels and observations were not independent as the null hypothesis would predict.

When inspecting the individual classification accuracies (see Table 4.1), we noticed that classification accuracy is below chance level for four participants. For those participants, it does not make sense to use activity in the F-ROI to distinguish between low and high levels of arousal. A possible explanation is that these participants did not follow the instructions, and were not genuinely trying to empathize with the IAPS pictures shown on the screen (as could be deducted from the log book in two cases). Because we did not want to exclude participants ad hoc based on results, they are still part of the data in the main paper. The results of the subsequent analyses on ad level were robust to removing the four cases with a classification accuracy below 50, and three cases with a classification accuracy equal to 50 (chance level).

| Descriptives | Classification accuracy in % |
| --- | --- |
| *M* | 55.10 |
| SD | 5.63 |
| Range | 45 - 65 |
| 95% CI | 53.12 - 57.08 |

**Table 4.1**

**Descriptives of Classification Accuracies across Participants**
